# Predictors of major complications after elective abdominal surgery in cancer patients

**DOI:** 10.1186/s12871-018-0516-6

**Published:** 2018-05-09

**Authors:** Claudia M. Simões, Maria J. C. Carmona, Ludhmila A. Hajjar, Jean-Louis Vincent, Giovanni Landoni, Alessandro Belletti, Joaquim E. Vieira, Juliano P. de Almeida, Elisangela P. de Almeida, Ulysses Ribeiro, Ana L. Kauling, Celso Tutyia, Lie Tamaoki, Julia T. Fukushima, José O. C. Auler

**Affiliations:** 1 Anesthesia Department, Instituto do Câncer do Estado de São Paulo, Av. Dr. Arnaldo, 251 - Cerqueira César, São Paulo, SP 01246-000 Brazil; 2 Anesthesia Department, Hospital das Clinicas da Faculdade de Medicina da Universidade de São Paulo, São Paulo, Brazil; 3 Universite Libre de Bruxelles, Hospital Erasmus, Bruxelles, Belgium; 4 Department of Anesthesia and Intensive Care, IRCCS San Raffaele Scientific Institute, Milan, Italy; 5 Vita-Salute San Raffaele University, Milan, Italy

**Keywords:** Perioperative complications, Surgery, Risk factors, Cancer, Critical care

## Abstract

**Background:**

Patients undergoing abdominal surgery for solid tumours frequently develop major postoperative complications, which negatively affect quality of life, costs of care and survival. Few studies have identified the determinants of perioperative complications in this group.

**Methods:**

We performed a prospective observational study including all patients (age > 18) undergoing abdominal surgery for cancer at a single institution between June 2011 and August 2013. Patients undergoing emergency surgery, palliative procedures, or participating in other studies were excluded. Primary outcome was a composite of 30-day all-cause mortality and infectious, cardiovascular, respiratory, neurologic, renal and surgical complications. Univariate and multiple logistic regression analyses were performed to identify predictive factors for major perioperative adverse events.

**Results:**

Of a total 308 included patients, 106 (34.4%) developed a major complication during the 30-day follow-up period. Independent predictors of postoperative major complications were: age (odds ratio [OR] 1.03 [95% CI 1.01–1.06], *p* = 0.012 per year), ASA (American Society of Anesthesiologists) physical status greater than or equal to 3 (OR 2.61 [95% CI 1.33–5.17], *p* = 0.003), a preoperative haemoglobin level lower than 12 g/dL (OR 2.13 [95% CI 1.21–4.07], *p* = 0.014), intraoperative use of colloids (OR 1.89, [95% CI 1.03–4.07], *p* = 0.047), total amount of intravenous fluids (OR 1.22 [95% CI 0.98–1.59], *p* = 0.106 per litre), intraoperative blood losses greater than 500 mL (2.07 [95% CI 1.00–4.31], *p* = 0.043), and hypotension needing vasopressor support (OR 4.68 [95% CI 1.55–27.72], *p* = 0.004). The model had good discrimination with the area under the ROC curve being 0.80 (95% CI 0.75–0.84, *p* < 0.001).

**Conclusions:**

Our findings suggest that a perioperative strategy aimed at reducing perioperative complications in cancer surgery should include treatment of preoperative anaemia and an optimal fluid strategy, avoiding fluid overload and intraoperative use of colloids.

**Electronic supplementary material:**

The online version of this article (10.1186/s12871-018-0516-6) contains supplementary material, which is available to authorized users.

## Background

In the last years, survival related to cancer has increased due to advances in diagnostic and therapeutic interventions, including implementation of management protocol (e.g. goal-directed hemodynamic therapy) in perioperative care [1–3]. Nowadays, surgery remains the cornerstone of treatment of solid neoplasms. Unfortunately, cancer patients are frequently characterized by a high perioperative risk because of immune system disturbances, decreased physiologic reserves, and long duration of procedures, with significant fluid and blood losses [1, 2]. Previous studies showed worse postoperative outcomes in this subgroup of patients, including higher mortality rates, increased costs and longer hospital and intensive care unit (ICU) length of stay [3–5]. Furthermore, perioperative complications negatively affect quality of life and may delay or preclude further cancer treatment, such as adjuvant chemotherapy. Therefore, preventing complications in this specific group of patients is of particular importance. Identification of predictive factors for complications is critical for several reasons. First of all, it allows a more accurate risk stratification and hence adequate planning of perioperative management. In addition, modifiable factors could become target for tailored interventions aimed at reducing postoperative complications. Finally, identification of predictive factors helps to identify areas for future investigations.

As of today, few studies focused on identifying predicting factors for perioperative complications in cancer patients undergoing abdominal surgery. Accordingly, we designed and performed a prospective observational study aimed at identifying predictive factors for major complications including mortality in patients undergoing elective abdominal surgery for cancer.

## Methods

We designed a prospective observational study including cancer patients undergoing elective abdominal surgery at the Cancer Institute of the University of Sao Paulo between June 2011 and August 2013. The Cancer Institute of the University of Sao Paulo is a 500-beds cancer referral centre, with about 6000 surgeries/year. The study was approved by the Ethics Committee (protocol number 283/10) and written informed consent obtained from every patient. The manuscript was prepared in accordance with the STROBE statement [6]. Approval from the EuroQol Group was also obtained for the use of the EQ-5D-3 L questionnaire [7].

Patients were included if they met all the following criteria: age greater than 18 years, diagnosis of a solid neoplasm, plan for elective open abdominal surgery with curative intent or bowel reconstruction from previous primary tumour excision, and written informed consent. The exclusion criteria were patient denial, emergency surgeries, participation in other studies and admission for palliative procedures.

### Anaesthetic and surgical management

All patients were anesthetised according to the standard institutional protocol for high-risk surgery. The general aspects of anaesthetic technique are described in detail in the (Additional file 1). Synthetic colloids were used (6% hydroxyethyl starch 130/0.4) in patients with no previous renal dysfunction, coagulopathy or infection.

### Data collection

Data were collected at baseline to describe baseline characteristics and demographics of patients, including comorbidities, health-related Quality-of-Life, oncological diagnosis and status of disease. Intraoperative data included type of surgery, therapeutic interventions and complications. A more detailed description of the collected data can be found in the (Additional file 1).

Patients were followed up daily after surgery for 30 days by four trained physicians, each with more than 2 years-experience of postoperative care. Clinical outcomes were evaluated during the intensive care unit (ICU) stay and then on the general ward.

In case of hospital discharge before day 30, a follow-up phone call was performed by two physicians. Those who conducted the follow-up telephone assessments had no participation in the perioperative care and had no access to the database. We used a standardized questionnaire to evaluate outcomes after hospital discharge, assessing clinical complications, vital status and quality of life.

Health related quality of life (HRQoL) was evaluated by the 5-dimensional scale of the EuroQoL (EQ-5D-3 L) rated with local utilities [8] and visual analogue scale (EQVAS) at 30 days after surgery. EQ-5D-3 L is a questionnaire with five dimensions regarding mobility, self-care, usual activities, pain/discomfort and anxiety/depression on three levels to define HRQoL values (utilities) for 245 health states. The visual analogue scale (VAS) used in the EQ-5D-3 L has two endpoints labelled ‘best imaginable health state’ and ‘worst imaginable health state’.

### Outcomes

Primary outcome was the composite endpoint of 30-day mortality and major complications defined as infectious, cardiovascular, respiratory, neurologic, renal and surgical complications. All complications were defined according to standard criteria (see [Additional file 1] for details on outcome definitions). Secondary outcomes were Health Related Quality of Life (HRQoL) 30 days after surgery, ICU and hospital length of stay.

### Statistical analysis

The results are expressed as the means with standard deviation (SD) or medians with interquartile ranges (IQRs) as appropriate. Normality was assessed with the Kolmogorov-Smirnov test. Univariate associations between potential risk factors and complications were assessed using the chi-square test, Fisher’s exact test, likelihood ratio test, t-test or Mann-Whitney U test. A forward multiple logistic regression analysis was then performed to estimate independent predictive factors for complications. This model included risk factors that were identified by univariate analysis (*p* < 0.05) plus the following clinically relevant data such as Karnofsky Performance Status Scale Score, presence of metastatic disease and HRQoL evaluation (utilities and VAS scores). To evaluate the stability of the effect estimates, bootstrap was applied for 1000 samples. All variables that remained significant after the bootstrap procedure were kept in the model. A receiver operating characteristic (ROC) curve was constructed, and the area under the ROC curve (AUC) determined to assess the discriminant ability of the multiple logistic regression models to predict complications.

A value of less than 0.05 was considered to indicate statistical significance (p), and all tests were two-tailed. Statistical analyses were performed using SPSS version 20 (SPSS, Inc., Chicago, IL, USA) and MedCalc for Windows, version 14.8.1 (MedCalc Software, Ostend, Belgium).

## Results

A total of 927 patients were assessed for eligibility (Additional file 1: Figure S1). After exclusions, a total of 308 patients were enrolled in the study.

Tables 1 and 2 describes baseline characteristics of the patients and type of performed surgical procedures. Most operations were colorectal and gynaecological procedures. Regional anaesthetic technique combined with general anaesthesia was performed in 80% of patients. A total of 166 (53.9%) patients were admitted to the ICU following surgery.

**Table 1:** Baseline characteristics of the patients and preoperative laboratory findings

Variable	Total	Complications		
		No	Yes	
	*n* = 308 (100%)	*n* = 202 (66%)	*n* = 106 (34%)	
Age (years), mean and standard deviation	59.7 ± 14.1	57.8 ± 14.5	63.3 ± 12.5	0.010
BMI (kg/m^2^), median (IQR)	25.6 (22.3–29.5)	25.9 (22.7–30.1)	25.3 (21.9–28.2)	0.066
Male gender	139 (45.1%)	87 (43.1%)	52 (49.0%)	0.316
Preoperative ambulatory reevaluation	31 (10.1%)	17 (8.4%)	14 (13.2%)	0.184
Functional status (<4METs)	76 (24.7%)	36 (17.8%)	40 (37.7%)	< 0.001
Systemic arterial hypertension	147 (47.7%)	93 (43.0%)	54 (51.0%)	0.413
Chronic obstructive pulmonary disease	34 (11%)	20 (9.9%)	14 (13.2%)	0.379
Tobacco use	29 (9.4%)	19 (9.4%)	10 (9.4%)	0.994
Coronary arterial disease	20 (6.5%)	8 (4.0%)	12 (11.3%)	0.013
Heart failure	25 (8.1%)	11 (5.4%)	14 (13.2%)	0.018
Diabetes mellitus	53 (17.2%)	29 (14.3%)	24 (22.6%)	0.067
Chronic renal disease	66 (21.4%)	32 (15.8%)	34 (32.0%)	0.001
Previous stroke	12 (3.9%)	5 (2.5%)	7 (6.6%)	0.075
Karnofsky Performance Status Scale Score (< 70%)	9 (2.9%)	4 (2.0%)	5 (4.7%)	0.283
Previous oncologic treatment	148 (48.1%)	95 (47.0%)	53 (50.0%)	0.620
Metastatic disease	55 (17.9%)	35 (17.3%)	20 (18.8%)	0.737
Preoperative abnormal EKG	67 (21.8%)	39 (19.3%)	28 (26.4%)	0.151
Hemoglobin (< 12 g/dL)	94 (30.5)	52 (25.7%)	42 (39.6%)	0.012
ASA physical status (≥3)	62 (20.1%)	28 (13.8%)	34 (32.0%)	< 0.001
Sodium (< 135 mEq/L)	16 (5.2%)	7 (13.5%)	9 (8.5%)	0.059
Potassium (< 3.5 or > 5.0 mEq/L)	34 (11%)	22 (11.0%)	12 (11.3%)	0.909
Systolic pressure (mmHg), median (IQR)	127 (112–140)	129 (113–142)	125 (111–138)	0.134
Diastolic pressure (mmHg), median (IQR)	80 (71–88)	80 (72–90)	80 (71–86)	0.194
Heart rate (bpm), median (IQR)	77 (67–89)	76 (67–89)	78 (67–89)	0.482
Creatinine (mg/dL), median (IQR)	0.84 (0.73–1.00)	0.82 (0.72–0.95)	0.87 (0.74–1.12)	0.020
Platelets (10^3^ cells/mm^3^), median (IQR)	234 (193–292)	233 (193–286)	239 (192–303)	0.686
EQ-5D-3 L	0.84 (0.72–0.88)	0.87(0.73–0.88)	0.81(0.67–0.88)	0.063
VAS	80 (60–90)	80(70–90)	70(60–87.5)	0.081

Pearson’s chi-square or Fisher exact test was used for categorical data. Normally distributed data are presented as the mean ± standard deviation and were analysed with the student t-test. Non normally distributed data are presented as the median (IQR) and were analysed with the Mann-Whitney test. *P* < 0.10 was considered for inclusion in the multiple logistic regression model. *BMI* body mass index, *EKG* electrocardiography, *ASA* American Society of Anesthesiologists, *IQR* interquartile range, *VAS* visual analogue scale, *EQ-5D-3 L* Health related quality of life scale – EuroQol

**Table 2:** Surgical characteristics of the study patients

Variable		Complications		
	Total	No	Yes	*P*
	*n* = 308	*n* = 202 (66%)	*n* = 106 (34%)	
Type of procedure				< 0.001
Colorectal surgery	100	66 (66%)	34 (34%)	
Hysterectomy and/or anexectomy	57	50 (88%)	7 (12%)	
Gastrectomy	27	17 (63%)	10 (37%)	
Liver resection	25	17 (68%)	8 (32%)	
Pancreatic surgery	22	11 (50%)	11 (50%)	
Exploratory laparotomy	22	16 (73%)	6 (27%)	
Bowel resection	13	10 (76.9%)	3 (23.1%)	
Esophagectomy	11	0 (0%)	11 (100%)	
Colon reconstruction	11	9 (81.8%)	2 (18.2%)	
Cystectomy	9	2 (22.2%)	7 (77.8%)	
Peritonectomy	5	0 (0%)	5 (100%)	
Biliary-enteric anastomosis	3	2 (66.7%)	1 (33.3%)	
Cytoreduction	3	2 (66.7%)	1 (33.3%)	

One hundred and six (34%) patients had at least one major complication (Table 3). A total of 32 patients (10.4%) had at least two complications. At 30 days, death occurred in 7 patients (2.3%), respiratory complications in 14 (4.5%), cardiovascular in 28 (9.1%), infectious in 17 (5.5%), renal in 40 (13.0%) and surgical in 29 (9.4%) patients (Table 4).

**Table 3:** Postoperative complications during the 30-day follow-up period

Outcomes	*n* (%)
Respiratory complications	14 (4.5%)
Acute respiratory failure	5 (1.6%)
Pneumonia	13 (4.2%)
Prolonged mechanical ventilation (> 48 h)	14 (4.5%)
Cardiovascular complications	28 (9.1%)
Acute myocardial infarction	4 (1.3%)
Cardiogenic shock	23 (7.5%)
Stroke	2 (0.6%)
Infectious complications	17 (5.5%)
Septic shock	14 (4.5%)
Severe sepsis	17 (5.5%)
Acute kidney injury	40 (13.0%)
Surgical complications	29 (9.4%)
Anastomosis dehiscence	12 (3.9%)
Operative wound dehiscence	3 (1.0%)
Surgical wound infection	11 (3.6%)
Re-operation	17 (5.5%)
Death	7 (2.3%)
Septic shock (pulmonary origin)	1 (0.3%)
Septic shock (abdominal origin)	4 (1.3%)
Septic shock (multiple origin)	2 (0.6%)

**Table 4:** Table describing intra-operative events and postoperative complications

Intraoperative variables			
Regional anaesthesia	159 (78.7%)	86 (81.1%)	0.617
Use of colloids (HES 6%)	96 (47.5%)	72 (68.0%)	0.001
Use of continuous vasopressors	4 (2.0%)	24 (22.6%)	< 0.001
Intraoperative fluid volume (L), median (IQR)	3.1 (2.5–4.5)	5.0 (3.5–6.5)	< 0.001
Red blood cell transfusion	7 (3.5%)	21 (19.8%)	< 0.001
Estimated bleeding (> 500 mL)	38 (18.8%)	50 (47.1%)	< 0.001
Surgical length (min), median (IQR)	247 (166–331)	315 (220–385)	< 0.001

Pearson’s chi-square or Fisher exact test were used for categorical data; non normally distributed data are presented as the median (IQR) and were analysed by the Mann-Whitney test. *P* < 0.10 was considered for inclusion in the multiple logistic regression model

*IQR* interquartile range

The group of patients who developed complications was older (63.3 ± 12.5 years vs. 57.8 ± 14.5 years, *p* < 0.001), had a higher prevalence of poor preoperative functional status, defined by less than 4 metabolic equivalents (METS) (37.7% vs. 17.8%, *p* < 0.001), had a higher prevalence of coronary artery disease (11.3% vs. 4.0%, *p* = 0.013), heart failure (13.2% vs. 5.4%, *p* = 0.018) and chronic kidney disease (32% vs. 15.8%, *p* = 0.001) (Table 1). The incidence of metastatic disease was similar between groups. The occurrence of preoperative anaemia (haemoglobin lower than 12 g/dL) was higher in the group with complications compared to the group without complications (39.6% vs. 25.7%, *p* = 0.012), as the number of patients with an ASA score equal to or greater than 3 (32.0% vs. 13.8%, *p* < 0.001).

There was no difference between groups in the preoperative Health Related Quality of Life (HRQoL) and in the VAS values (Table 1).

Patients who developed major complications, when compared to patients without complications, were more likely to receive colloids (68.0% vs. 47.5%, *p* = 0.001), to receive vasopressors due to hypotension (22.6% vs. 2.0%, *p* < 0.001), and higher amount of intravenous fluid [5.0 L (IQR 3.5–6.5) vs. 3.1 L (2.5–4.5), *p* < 0.001] intra-operatively. Additionally, patients who developed major complications had a higher incidence of intraoperative bleeding (47.1% vs. 18.8%, *p* < 0.001) and were more exposed to red blood cell transfusions (19.8% vs. 3.5%, *p* < 0.001) and had longer duration of surgery [315 min (220–385) vs. 247 min (166–331), *p* < 0.001) (Table 4). The group that required ICU and developed major complications had longer length of ICU stay [3.8 days (IQR 2–4 days) vs. 1.8 days (IQR 1–2 days), *p* < 0.001) and of hospital stay [12.8 days (IQR 8–15 days) vs 6.9 days (IQR 6–8 days), *p* < 0.001) compared to patients without complications (Additional file 1: Figure S2a and b).

In a sample of 191 patients, health-related quality-of-life decreased in both groups. On the other hand, VAS did not change (Table 5).

**Table 5:** Health-related quality-of-life evaluated through EQ-5D-3 L and VAS

	Preoperative (*n* = 191)	Postoperative (*n* = 191)	P*
EQ5D	0.87 (IQR 0.78–1.00)	0.83 (IQR 0,72–0,88)	0.001
VAS	80 (IQR 70–90)	80 (IQR 70–90)	0.577

*Wilcoxon test, *p* < 0.05 was considered significant

EuroQoL (EQ-5D-3 L) rated with local utilities and visual analogue scale (VAS)

In the forward multiple logistic regression model, age, ASA physical status, preoperative anemia, use of colloids, blood losses and hypotension requiring intraoperative vasopressors were independently related to complications (Table 6).

**Table 6:** Results of multiple logistic regression analysis to determine predictive factors for major complications following cancer surgery

Variable	Odds ratio (CI 95%)	*P*
Age (years)	1.03 (1.01–1.06)	0.010
ASA physical status (≥3)	2.61 (1.34–5.49)	0.004
Preoperative hemoglobin (< 12 g/dL)	2.13 (1.15–4.15)	0.016
Use of colloids	1.89 (0.99–3.75)	0.049
Estimated blood loss (> 500 mL)	2.06 (0.97–4.42)	0.048
Intraoperative hypotension requiring vasopressor	4.67 (1.41–15.48)	0.004

*ASA* American Society of Anesthesiologists

*p* < 0.05 was considered significant

A discriminant analysis identified an area under the ROC of 0.799 (95% CI 0.747–0.851, *p* < 0.0001) (Additional file 1: Figure S3). In order to verify if the identified independent predictors could be applied to the different subsets of surgical procedures, the model was applied to surgeries with colonic resection from the case mix and the ROC curve was analysed (AUC 0.798 [CI95% 0.749–0.842]) with no significant differences found when compared with the general abdominal case-mix (*p* = 0.87).

Applying model-based resampling with bootstrap for 1000 samples, after adjusting for all variables included in the model, independent predictors were age (odds ratio [OR] 1.03 [1.01–1.06], *p* = 0.010), ASA physical status equal to or greater than 3 (OR 2.61 [1.34–5.49], *p* = 0.004), a preoperative haemoglobin level lower than 12 g/dL (OR 2.13 [1.15–4.15], *p* = 0.016), the intraoperative use of colloids (OR 1.89, [0.99–3.75], *p* = 0.049), estimated bleeding greater than 500 mL (OR 2.06 [0.97–4.42], *p* = 0.048), and hypotension requiring vasopressors (OR 4.67 [1.41–15.48], *p* = 0.004) (Table 6). Compared to the initial model, only the increased amount of intraoperative fluids (OR 1.22 [0.99–1.60], *p* = 0.097) after adjusting for everything else in the model was not an independent predictor for complications. In the discriminant analysis of the model with independent predictors after bootstrap the area under the ROC curve was 0.782 (95% CI 0.728–0.836, *p* < 0.0001) (Additional file 1: Figure S2). Comparing the area under the curves from both models, the difference was not significant between them (*p* = 0.052).

## Discussion

In our prospective observational study, we found that, in cancer patients undergoing abdominal surgery, age, ASA score, preoperative anaemia and intraoperative bleeding, use of colloids, higher amount of fluids and vasopressors were identified as predictors of major postoperative complications including mortality. Excluding age and ASA score, all of the factors identified in our study are modifiable. These findings suggest that a perioperative strategy based on the treatment of preoperative anaemia, implementation of conservative blood management and effective bleeding control and haemodynamic management during surgery may improve outcomes in patients undergoing elective major oncologic abdominal surgery.

Preoperative anaemia has been associated with worse outcomes in surgical patients [9]. Anaemia in cancer patients is common and multifactorial. Blood loss, decreased bone marrow production, increased destruction of red blood cells and drug toxicities are involved in cancer-related anaemia [10]. Wu et al. [11] reported in a retrospective study that preoperative anaemia was associated with postoperative 30-day mortality and cardiovascular events in patients undergoing major non-cardiac surgery. Carson et al. [12] also reported an increased risk of death and cardiovascular complications in the postoperative period in patients with preoperative anaemia, particularly in patients with previous cardiovascular disease. Dunne et al. [13] in a prospective study demonstrated that low preoperative haematocrit levels were associated with an increased incidence of pneumonia, hospital length of stay and mortality. Moreover, preoperative anaemia is also a known risk factor for postoperative anaemia and increased requirements for perioperative blood transfusion, which contribute to postoperative complications [9, 14, 15]. Preventive strategies for patients with preoperative anaemia may enhance postoperative outcomes [15]. The use of preoperative recombinant human erythropoietin in cancer patients appears to be safe, although previous studies have associated its use with the progression of disease and mortality [16, 17]. A recent pilot study in patients undergoing cardiac surgery suggested that preoperative red blood cell transfusion reduced the intraoperative requirements for additional transfusion and decreased postoperative organ failure [18]. Despite all complications related to transfusion in cancer patients undergoing abdominal surgeries, this group of patients may benefit from postoperative liberal transfusion strategies, as showed by Almeida et al. [19].

Fluid overload in the perioperative period was associated with an increased rate of severe postoperative complications in the initial model in our study, but it was excluded after bootstrap due to sampling variation, Nevertheless, this factor points out as an important standard of care to prevent postoperative complications in general surgical patients. Excessive positive fluid balance can lead to interstitial oedema, microcirculatory impairment, tissue hypoxia, chloride overload, organ failure and death [20–22]. Previous studies have demonstrated that patients undergoing major abdominal surgery can benefit from a restrictive strategy of fluid management in the perioperative period. Different studies [23–25] reported that a restrictive strategy can reduce the incidence of severe complications. Our group reported that a mean positive fluid balance greater than 1100 mL/24 h within the first 72 h after ICU admission was an independent predictive factor for mortality in cancer patients [26]. Half of these patients were surgical patients in the postoperative period after major surgery for cancer treatment. Thus, these findings suggest that a perioperative goal-directed therapy with a restrictive fluid management may prevent fluid overload and postoperative complications. The recently completed Restrictive versus liberal fluid therapy in major abdominal surgery (RELIEF) multicentre randomized trial will hopefully provide definitive clues on this issue [27].

This study also identified that the use of the colloids starches in the intraoperative period is an independent factor associated with postoperative complications. Although it was not possible to establish causal effects because of our study design, recent studies have shown an increased risk of renal dysfunction and mortality in patients with sepsis related to the use of artificial colloids such as starches [28, 29]. Myburgh et al. compared the use of crystalloid with hydroxyethyl starch (HES) 130/0.4 in critically ill patients, including patients after elective and emergency surgery, in a large randomized controlled trial [30]. This trial showed higher incidence of adverse events including acute kidney injury in patients who received HES as compared to saline. Therefore, the current safety concern regarding the use of HES is justified, and evidence is needed before hypothesizing their use for surgical patients [31–35].

Hypotension requiring a vasopressor during surgery was identified in our study as an independent factor predicting a poor outcome after surgery. Cancer surgery is often characterized by long and extensive resection with excessive blood losses, ischemia and reperfusion injuries and exposure to blood transfusion. All of these factors can lead to an exacerbated systemic inflammatory response syndrome (SIRS) and vasodilatory shock. SIRS-related cardiovascular failure has been shown to be associated with postoperative organ failure, severe complications and mortality [36]. Another important finding of our study was the association of bleeding greater than 500 mL with postoperative complications. Excessive blood losses can lead to organ failure due to an imbalance of oxygen delivery, tissue hypoxia and inflammation. Moreover, significant intraoperative blood losses are a significant risk factor for postoperative anaemia and the need for red blood cell transfusion, both of which are associated with an increased rate of severe postoperative complications, an increased length of hospital stay and mortality [37, 38].

Our study reported that older age and a higher ASA status were independent characteristics associated with postoperative complications. The relationship between age and co-morbidities with poor outcomes after surgery has been well described in previous studies [39]. The worldwide demographic transition, particularly in developing countries, has led to an increase in the number of patients older than 65 years undergoing major surgery, with higher operative mortality [40]. In our study, the mean age of the group of patients with severe complications was 63.3 years-old, which was 7 years older than the group without complication. The physiologic reserve declines with age and explain why older patients have a reduced tolerance for major oncologic resections.

Our study has some limitations. First, it was a single centre study performed in a tertiary referral centre for cancer treatment and our findings may be related to the characteristics of our elective surgical population and therefore not necessarily generalizable to other centres. Second, our study included a case-mix of abdominal surgeries that may have specific outcomes, although the operative severity itself was not identified as being an independent predictor of complications. There may be a benefit of evaluating such a case mix for anaesthetists, because even in tertiary centres abdominal surgeries have a standard approach. The patients who had severe complications were sicker and older than those who did not. Therefore, the causal effect of these factors cannot be confirmed by our results.

## Conclusions

In conclusion, this study suggests that a higher age and ASA score, preoperative anaemia, intraoperative blood losses, intraoperative hypotension requiring vasopressors, and the administration of artificial colloids in patients undergoing major abdominal cancer surgery were independently associated with severe complications and death. These findings suggest that a perioperative management strategy based on the treatment of preoperative anaemia, implementation of haemostatic surgical techniques, conservative blood management, and adequate hemodynamic control avoiding hypotension might reduce severe complications in this population. Future randomised studies aiming at improving perioperative outcome in cancer patients should focus on optimizing these aspects.

## Additional file

Additional file 1: Supplementary Appendix. (DOCX 179 kb)

## Abbreviations

ASA: American society of anesthesiologists
AUC: Area under the ROC curve
EQ-5D-3 L: EuroQoL
HES: Hydroxyethyl starch
HRQoL: Health related quality of life
ICU: Intensive care unit
METS: Metabolic equivalents
OR: Odds ratio
RELIEF: Restrictive versus liberal fluid therapy in major abdominal surgery
ROC: Receiver operating characteristic
SD: Standard deviation
SIRS: Systemic inflammatory response syndrome
VAS: Visual analogue scale
