# Supplementary material for: Predictors of major complications after elective abdominal surgery in cancer patients
Source: BMC Anesthesiol. 2018 May 9;18:49. doi: 10.1186/s12871-018-0516-6 (PMC5944034; doi:10.1186/s12871-018-0516-6)
Supplement: Supplementary file 1 — Supplementary Appendix. (DOCX 179 kb) [file 12871_2018_516_MOESM1_ESM.docx]

**Additional File 1**

**ELECTRONIC SUPPLEMENTARY MATERIAL**

**Predictors of Major Complications after**

**Abdominal Surgery in Cancer Patients**

*Claudia Marquez Simões, Maria José Carvalho Carmona, Ludhmila Abrahão Hajjar, Jean-Louis Vincent, Giovanni Landoni, Alessandro Belletti, Joaquim Edson Vieirab, Juliano Pinheiro de Almeida, Elisangela Pinto de Almeida, Ulysses Ribeiro Jr, Ana Laura Kauling, Celso Tutyia, Lie Tamaoki, Julia Tizue Fukushima, José Otávio Costa Auler Jr*

**TABLE OF CONTENTS**

**Supplementary Methods** page 2

**Figure S1** page 6

**Figure S2a/2b** page 7

**Figure S3** page 8

**Supplementary References** page 9

**SUPPLEMENTARY METHODS**

*Anaesthetic technique*

Anaesthesia was induced with fentanyl (3-5 μg/kg) or sufentanil (0.3-0.5 μg/kg), midazolam (0.03-0.05 mg/kg), etomidate (0.2-0.3 mg/kg) or propofol (2 mg/kg) and cisatracurium (0.2 mg/kg). Anaesthesia was maintained either with inhalational agents (isoflurane or sevoflurane) in oxygen with opioids as needed or intravenously with propofol. Regional anaesthesia (an epidural or subarachnoid anaesthesia) was performed before surgery to provide effective analgesia of the patient’s wound. Intraoperative loading with a local anaesthetic (bupivacaine or ropivacaine) and morphine or with an infusion of local anaesthetic and an opioid was performed according to institutional protocols.

After tracheal intubation, all patients received invasive mechanical ventilation with intermittent positive pressure with a tidal volume of 6-8 mL/kg, positive end-expiratory pressure of 5 to 8 cm H_2_O, and fraction of inspired oxygen (FiO_2_) of 0.4 to 0.6 to maintain arterial oxygen saturation above 95%.

During surgery, most patients were monitored with a central venous line and indwelling radial artery catheter. At the anesthesiologist’s request, the cardiac index was obtained from an arterial pulse contour analysis (Vigileo, FloTracSensor, Edwards Lifesciences, Irvine, CA 92614 USA). Fluid management and the administration of a vasopressor and inotropic agents were performed to maintain a mean arterial pressure of 65 mmHg or higher, a central venous pressure of 8-12 mmHg, urinary output higher than 0.5 mL/Kg/h, oxygen venous saturation equal to or greater than 70% and cardiac index equal or higher than 2.2 L/min/m^2^. Synthetic colloids were used (6% hydroxyethyl starch 130/0.4) in patients with no previous dysfunction, no coagulopathy and without any infection. After surgery, if the patients had hemodynamic instability, were on mechanical ventilation, or had a presumed high risk of postoperative complications, they were admitted to the intensive care unit (ICU). If these factors were not present, the patients were discharged to a regular ward after anesthetic recovery.

*Data collection*

Preoperatively collected data included age, gender, weight, height, body mass index (BMI) and comorbidities (diabetes mellitus, hypertension, coronary artery disease, chronic kidney disease characterized by glomerular filtration rate of less than 60 mL/min/1.73 m^2^, chronic heart failure, chronic obstructive pulmonary disease, and previous stroke. The type of tumor, previous oncologic treatments (surgery, chemotherapy or radiotherapy), the presence of metastatic disease and the Karnofsky performance status were also recorded. Preoperative laboratory data included leukocyte and platelet counts and a measurement of hemoglobin, creatinine, and electrolytes. Preoperative ASA (American Society of Anesthesia) scores were recorded for all patients.

Intraoperative data were collected, including the type of procedure, the length of anesthesia and surgery, the amount of intraoperative fluid replacement, the type of fluids administered (crystalloid or colloids), urine flow, estimated blood loss, intraoperative transfusion, hemodynamic instability, hypotension and the administration of vasoactive drugs.

During the 30 days of postoperative follow-up, clinical data were collected daily by 4 trained physicians with more than 2 years experience in caring for postoperative patients. Clinical outcomes were evaluated during the ICU stay and in the regular ward. Operative mortality was considered as death from all causes in the 30 days after surgery. For patients discharged from hospital before day 30, a telephone follow-up call was performed by two independent and blind physicians with no access to database and perioperative care.

### Outcome definitions

### During the 30 day postoperative period, major complications were evaluated, including mortality due to all causes. The severe complications included cardiovascular complications, respiratory, renal, infectious and surgical complications.

Respiratory complications were defined as prolonged need for mechanical ventilation (longer than 48 hours), pneumonia or acute respiratory failure. Pneumonia was diagnosed if patient the patient had a new, persistent, or progressive lung infiltrate according to the chest radiograph and if at least 2 of the following criteria were present: a temperature of 38°C or higher, leukocytosis greater than 12,000 cells/μL or leukopenia less than 3,000 cells/μL, or purulent endotracheal secretions with a Gram stain showing more than 25 neutrophils and fewer than 10 epithelial cells per field.^1^ Acute respiratory failure was defined by standard criteria.^2^

Cardiovascular complications were defined as acute myocardial infarction, cardiogenic shock or stroke. An electrocardiogram was performed twice daily for patients in the ICU stay and accordingly to clinical judgment on the regular ward. Perioperative cardiac ischemia was considered if the creatine kinase MB level was elevated to at least 5 times the upper limit of normal (>30 ng/mL), if troponin I values were greater than 5 ng/mL during the first 72 hours, if new pathological Q waves appeared, if coronary artery occlusion was angiographically documented, or if there was imaging evidence of new loss of viable myocardium.^3^ Cardiogenic shock was defined as the presence of tachycardia, hypotension, and poor perfusion associated with venous central saturation (ScVO_2_) less than 65% or metabolic acidosis (increase in the base deficit >4) or an increase in lactate level (>3 mmol/L) in the absence of a cause other than heart failure.^4^ Stroke was characterized by a new focal deficit with a compatible image on computed tomography.

Infectious complications included severe sepsis and septic shock, defined according to the Surviving Sepsis Caimpaign guidelines.^5^

Renal complications were defined as acute kidney injury (AKI) stage 2 or 3 according to Acute Kidney Injury Network (AKIN) criteria.^6^

Surgical complications included reoperation, wound infection, and surgical wound or anastomosis dehiscence.

**Figure S1 –** Study flow.

Enrollment

Analysis

Excluded from analyses (n=0)

Analyzed (n=308)

Follow-up

Lost to follow-up (n=20)

- No telephone or no response in the follow-up period (n=13)
- Remote locations and no return to the institutional follow-up (n=7)

**Excluded (n=575)**

**Not meeting inclusion criteria (n= 35)**

- Declined to participate (n=18)
- Others (n=17)

**Meeting exclusion criteria (n= 540)**

- Not reported cardiovascular outcomes (n=147)
- Not reported renal complications (n=131)
- Enrolled in interventional studies (n=120)
- Not reported surgical complications (n=98)
- Others (n=44)

Enrolled (n=328)

Assessed for elegibility (n=927)

**Figure S2a** – Intensive Care Unit (ICU) length of stay of postoperative non-complications group (n=202) compared to complications group (n=106).

**Figure S2b** – Hospital length of stay of postoperative non-complications group (n=202) compared to complications group (n=106).

**Figure S3 -** Receiver-operating characteristic (ROC) curve from multiple logistic regression model related to postoperative complications in elective abdominal surgeries in cancer patients.


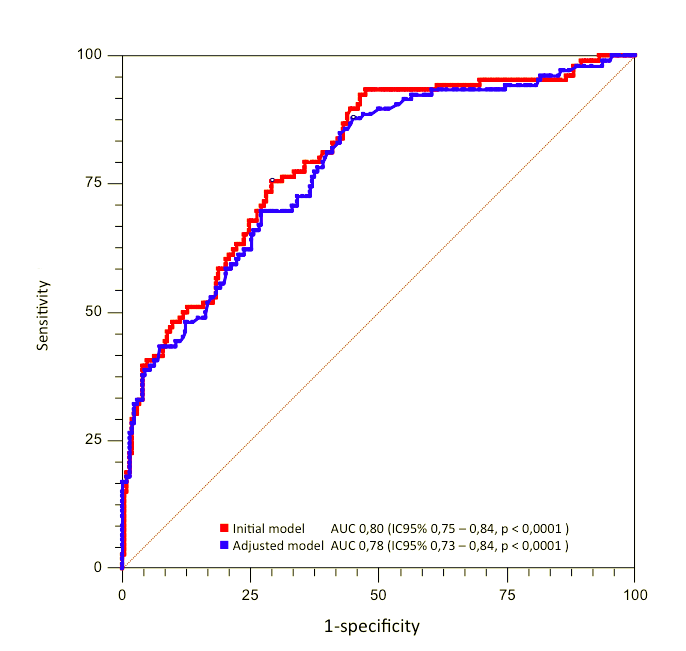


**SUPPLEMENTARY REFERENCES**

1. Croce MA. Postoperative pneumonia. *Am Surg*. 2000;66:133-137.

2. Burt CC, Arrowsmith JE. Respiratory failure. *Surgery*. 2009;27:475-479.

3. Thygesen K, Alpert JS, White HD; Joint ESC/ACCF/AHA/WHF Task Force for the Redefinition of Myocardial Infarction. Universal definition of myocardial infarction. *Eur Heart J*. 2007;28:2525-38.

4. Massé L, Antonacci M. Low Cardiac Output Syndrome: Identification and Management. *Crit Care Nurs Clin North Am*. 2005;17:375-83, x.

5. Dellinger RP, Levy MM, Rhodes A, et al. Surviving Sepsis Campaign: International Guidelines for Management of Severe Sepsis and Septic Shock: 2012. *Crit Care Med.* 2013;41:580-637.

6. Mehta RL, Kellum JA, Shah SV, et al. Acute Kidney Injury Network: report of an initiative to improve outcomes in acute kidney injury. *Crit Care*. 2007;11:R31.
